# Genetic modification of chicken germ cells

**DOI:** 10.1111/j.1749-6632.2012.06744.x

**Published:** 2012-10-10

**Authors:** Tae Sub Park, Jae Yong Han

**Affiliations:** Department of Agricultural Biotechnology, Research Institute for Agriculture and Life Sciences, College of Agriculture and Life Sciences, Seoul National UniversitySeoul, Korea

**Keywords:** primordial germ cell, germline chimera, transgenesis, avian biotechnology

## Abstract

Over the past two decades numerous reports have demonstrated that the genetic modification of poultry genomes has great potential for improving poultry production; moreover, it may be used as a powerful tool for the production of industrial proteins. To date, transgenic techniques have been established for generating transgenic birds that express recombinant human proteins in hen eggs, as well as tissue-specific genes as an animal model. The production of transgenic birds is a promising approach that could have practical applications in agriculture and biopharmacology, in addition to advancing our understanding of avian biology. Finally, germ cell–mediated transgenesis could provide a more efficient strategy for creating gene-targeted insertions and deletions in avian species.

## Introduction

Chick embryos are an excellent and reproducible model system for research into embryonic development.^1^ The creation of an efficient technique for producing transgenic chicks may lead to industrial applications in agriculture and biopharmacy; moreover, it will advance our understanding of avian biology itself. Numerous reports have demonstrated that the genetic modification of poultry genomes has great potential for improving poultry production and will provide a powerful tool for the production of industrial proteins. However, despite the potential for genetic manipulation, the virus-independent transgenesis procedure established in mammals cannot be applied to avian species owing to physiological and developmental differences between these groups. Furthermore, it is difficult to modify the genome of avian germ cells through conventional gene transfer. To date, retroviral and lentiviral transduction techniques have been established to generate transgenic birds; however, despite the availability of a valid technique for viral transduction for the creation of transgenic birds, many obstacles exist for realizing its practical applications due to relatively low and variable rates of germline transmission and transgenic offspring production, as well as safety issues related to viral usage. Thus, the generation of transgenic poultry by nonviral integration should be a prerequisite for the safe introduction of biotechnology to practical applications.

In “The Chick: A Great Model System Becomes Even Greater,” Stern^2^ stated: “Now, it has become even more powerful thanks to several new technologies: *in vivo* electroporation, ES cells, novel methods for transgenesis …. In combination with classical techniques such as grafting and lineage tracing, the chicken is now one of the most versatile experimental systems available.” Currently, chick models play a pivotal role in animal research as an alternative and outbreed experimental species to humans. Thus, methods for chicken transgenesis that are based on nonviral and viral integration should be advanced. Furthermore, transgenic techniques are not only applicable to experimental models but could also be used to design industrial applications.

## Germ cells

In 1997, Dolly the sheep was cloned from a somatic cell by nuclear transfer.^3^ Although animal cloning by somatic cell nuclear transfer has been successful and this technical advance has changed the basic paradigm of reproductive biology, germ cells are the only cell lineage that can transfer whole genetic material to the next generation. In sexual reproduction, two haploid germ cells (a sperm cell from the male parent and an oocyte from the female parent) fuse at fertilization, producing a single-celled zygote that develops through a series of embryonic developmental stages into a full-term offspring. Thus, germ cells are the most important cell type for maintaining a species. Additionally, germ cells are closely related to various birth defects and germ cell tumors, including ovarian and testicular cancers.

In mice, the regulatory process and inductive mechanism for germ cell specification have been extensively investigated. The mouse germ cell lineage is segregated from the pluripotent epiblast during implantation.^4^ Germ cell specification requires autonomous signaling by bone morphogenetic protein 4 secreted from extraembryonic ectoderm and visceral endoderm near the proximal epiblast.^5^,^6^ The newly derived germ cells from the epiblast (primordial germ cells (PGCs)) are localized primarily outside of the embryo and then migrate through the hindgut endoderm toward the developing genital ridges. At the end of their migration into the genital ridges, mouse germ cells undergo significant epigenetic reprogramming during proliferation and differentiation. At this developmental stage, the erasure of CpG DNA methylation at imprinted genes in both male and female germ cells and reactivation of the inactive X chromosome in females occur.^7^,^8^ In humans, because of ethical constraints and accessibility, studies of human germ cell differentiation are limited. Thus, alternative cell-based approaches and animal models are necessary for further study of human germ cells. Derivation of human germ cells from human embryonic stem cells (ES cells) could be one of the promising approaches for studying human germ cell differentiation *in vitro.*^9^,^10^

In birds, PGCs localize initially to the central zone of the zona pellucida in undifferentiated embryos at stage X and then migrate into the developing gonads through embryonic blood vessels. This migration route differs from that in mammals; however, little is known about germ cell specification and its mechanism(s) in avian species. The predetermined model, seen in *Caenorhabditis elegans* and *Drosophila*, in which the germplasm is a critical determinant of the germ cell lineage, is distinct from the induced model seen in mammals, in which inductive signaling is necessary for germ cell lineage specification. Tsunekawa *et al*.^11^ demonstrated that chicken vasa (cVASA) protein was predominantly localized to granulofibrillar structures surrounding the mitochondrial cloud in oocytes, and they suggested that this cVASA-containing cytoplasmic structure is a precursor to the germplasm in chickens. However, there is no definite evidence for germ cell segregation in chickens. Thus, we characterized avian PGCs at early embryonic stages and germ cells in adult testis, and investigated chicken germline development through an analysis of specific transcript and protein expression. Recently, an *in vitro* culture system for chicken PGCs was established^12^,^13^ and a microRNA regulatory network governing pluripotency of the undifferentiated blastoderm at stage X and germ cell differentiation in chickens was reported.^14^

## Germline modification

Genetically modified animals have enormous value in agriculture and medicine, as well as in basic research. An improved understanding of the basic processes governing germ cell and embryo development will enable us to efficiently generate transgenic model animals for studying infertility, birth defects, and human disease. Furthermore, increased knowledge about germ cells and germline modification will enable technical applications in industry.

For germline modification in vertebrates, including mammals and aves, various transgenic strategies have been developed. To create transgenic aves, a viral transduction technique using a retrovirus or lentivirus has been established. Conventional virus-mediated transgenic procedures involve the transduction of undifferentiated blastodermal cells at stage X by injecting concentrated replication-deficient virus. Recently, transgenic hens produced by the injection of a lentivirus carrying a recombinant human interferon (rhIFN) gene under the control of a synthetic tissue-specific promoter into the blastoderm were generated.^15^ These transgenic hens produced rhIFN at concentrations of 3.5–426 μg/mL in their eggs. To evaluate the functional reactivity of recombinant proteins deposited in transgenic hen eggs, Komada *et al*.^16^ produced transgenic hens expressing human erythropoietin, which has N- and O-linked glycosylation patterns. Also, Kwon *et al*.^17^ recently demonstrated the production of a biofunctional recombinant human interleukin 1 receptor antagonist (rhIL-1RN) in lentivirus-mediated transgenic quail. Although a viral transduction technique for generating transgenic birds has been established and verified, there are several obstacles to its practical application due to relatively low rates of germline transmission and transgenic offspring production, as well as safety issues associated with the usage of a viral vector. Based on the adaptation of procedures used for mammals, nonviral methods for transgenesis such as sperm-mediated gene transfer^18^,^19^ and the direct microinjection of DNA into the blastodisc^20^ have been developed. However, these protocols are laborious, exhibit low efficiency, and require the sacrifice of a large number of hens to collect fertilized eggs.

Recently, van de Lavoir *et al*.^21^ genetically modified chicken PGCs by the electroporation of a nonviral expression vector to produce transgenic offspring through germline transmission. However, the frequency of transgene integration into the genome as well as the rate of gene transfer into germ cells remain insufficient for generating transgenic birds using virus-independent conventional methods. For efficient transgene integration into the genome of chicken PGCs, Leighton *et al*.^22^ used phiC31 integrase and specific elements. phiC31 integrase catalyzes site-specific recombination between an attB site and an attP site; thus, the co-transfection of an integrase and attB-containing plasmid could improve genomic insertion into chicken PGCs. They showed increased frequencies of transgene integration into endogenous pseudo attP sites in the chicken PGC genome when phiC31 integrase was co-introduced; however, there is no report of the production of transgenic chickens using phiC31 integrase and an attB element. In addition, the *in vitro* and *in vivo* silencing of transgene expression following nonviral transfection has hampered the stable expression of antibiotic genes for selection and specific expression in target tissues.^21^–^23^ Leighton *et al*.^22^ demonstrated that the usage of phiC31 integrase and an attB element could be an efficient tool for genomic insertion; however, they also reported the transcriptional silencing of a transgene in chicken PGCs even after the co-transfection of phiC31 integrase and attB sequences. We previously showed that the methylation of a transgenic promoter in the transgenic chicken could lead to transgene transcriptional silencing in a tissue-specific manner *in vivo*, although little is known about the control of gene expression in avian species via DNA methylation.^23^ To overcome this transcriptional repression, HS4 insulator, which is the first insulator identified in vertebrates, derived from the 5′ end of the chicken β-globin locus, has been used in chicken PGCs.^21^

Based on the latest knowledge in the field, transgenic techniques have been rapidly and dramatically advanced. The use of transposons and transposase is one promising method for transgenesis through stable transgene expression without tissue-specific repression, as well as efficient transgene insertion into genomic structures in species from insects to mammals. Since the first DNA transposon was identified in maize,^24^ various transposon elements and transposases have been used for the genetic modification of several organisms. Transposons are genetic elements that can relocate between different genomic sites, and the enzyme transposase can excise unique DNA sites and recombine transposons into targeted sites in the genome. One advantage of the transposon elements such as *piggyBac* and Tol2 system is virus-independent gene transfer. As mentioned above, viral gene transfer carries safety issues for industrial applications; however, nonviral transposons could be safely and efficiently applied for various purposes.

**Figure 1:**
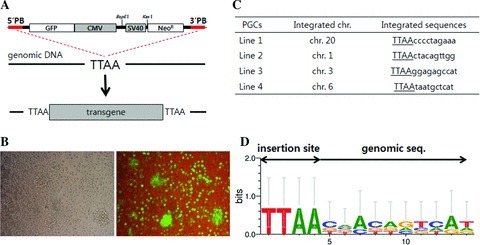
(A) *piggyBac* CMV-GFP vector map and integration site in genomic DNA. The *piggyBac* transposon was inserted into TTAA sequences by a cut-and-paste mechanism. (B) GFP-expressing chicken primordial germ cells (PGCs). After transfection and G418 selection, chicken PGCs stably and strongly expressed GFP. (C) The integrated chromosomes and sequences in chicken PGCs. The *piggyBac* transposon can integrate into various chromosomes without any preference in chicken cells. (D) WebLogo image of the inserted sequences in chicken PGCs. Only the TTAA site was conserved; no other unique sequence was found (WebLogo 3; http://weblogo.threeplusone.com).

Among DNA transposons, the *piggyBac* transposon identified from the cabbage looper moth *Trichoplusia ni*^25^ was found to efficiently transpose a transgene in a chicken cell line as well as in mice and humans.^26^–^28^*piggyBac*-mediated gene transfer enhanced the germline transmission efficiency of a transgene in mice^29^ and successfully reprogrammed completely differentiated mouse and human fibroblasts into induced pluripotent stem cells.^30^ Interestingly, *piggyBac* integration sites ascertained from mouse and human cell lines as well as insects presented no obvious consensus sequences for DNA recombination; only TTAA tetranucleotides are required for an integration sequence (Fig. 1A).^26^–^28^ The unique cut-and-paste mechanism of the *piggyBac* transposon can rearrange a transgene based on the sequence TTAA in the genome, regardless of the species.^28^,^31^ Excision of the well-known transposon sleeping beauty generates a mutation that includes a 3-bp addition to a TA element, generating a 5-bp insertion.^27^ In contrast, the *piggyBac* transposon is frequently integrated into genomic DNA without creating an insertion or deletion. Additionally, the genomic integration sites of the *piggyBac* transposon are generally located in introns, regulatory regions, and repetitive units, and in intergenic areas without any defined transcriptional units. Thus, the *piggyBac* transposon could be more applicable for transgenesis because transgene insertion into any functional gene could lead to cell death and the retardation of transgenic embryo development. Lu *et al*.^28^ adapted *piggyBac*-mediated gene transfer in chick embryos by *in ovo* electroporation and demonstrated that it was a versatile transgene expression technique for chick embryos. Additionally, Sato *et al*.^32^ also applied Tol2 transposon element to conditionally express transgene during chick embryo development. They showed that Tol2 transposon was also useful for stable integration into genome and tetracyclin-dependent expression in chicken.

In this year, Macdonald *et al*.^33^ reported the application of *piggyBac* and Tol2 transposon elements to modify the genome of chicken PGCs. Through genome-wide analysis of transposon insertions, they found that transposons were dominantly integrated into intronic and intergenic regions of chicken genome. Subsequently, the viable transgenic chick has been produced using transposon element.^33^ We also established a transgenic production protocol based on a nonviral system for the genomic modification of chicken PGCs and the creation of transgenic birds using the *piggyBac* transposon and germline-competent chicken PGCs.^34^ After *piggyBac*-mediated green fluorescent protein (GFP) gene transfer into chicken PGCs and G418 selection, chicken PGCs expressed GFP constantly without transgene silencing (Fig. 1B). Based on DNA walking analysis, the insertion sites of the *piggyBac* transposon in various chicken PGC lines were conserved with TTAA target sequences (Figs. 1C and 1D). Finally, we created transgenic chickens using *piggyBac*-mediated gene transfer into chicken PGCs. Surprisingly, in transgenic chickens derived from donor PGCs with a *piggyBac* GFP transgene, various organs such as intestine, heart, and liver constantly expressed GFP without tissue-specific transgene silencing. Furthermore, the efficiency of germline transmission of donor PGCs even after *piggyBac* transposition ranged from 90–98% (95.2% on average).^34^ Compared to previous data, these transmission rates were higher and very uniform. In the first report on germline chimera production using established PGCs,^21^ the efficiency of donor PGC-derived chicks from germline chimeric chickens varied from 0.1–86.0%. Choi *et al*.^12^ reported 12.5–82.6% (49.0% on average) germline transmission, and Macdonald *et al*.^13^ estimated that the frequency of donor-derived sperm was 1–30% in the semen of recipient roosters.

A germline-competent chicken PGC line was established with a high efficiency of transmission to offspring, and *piggyBac* transposition into chicken PGCs improved the efficiency of transgenic chicken production and led to a high level of transgene expression.^33^–^35^ This nonviral technique for producing transgenic poultry is a promising approach that may be the best method for studying germ cell biology and could be useful for future practical applications.
